# Factors associated with the rates of coronary artery bypass graft and percutaneous coronary intervention

**DOI:** 10.1186/s12872-019-1264-3

**Published:** 2019-11-29

**Authors:** Agnus M. Kim, Jong Heon Park, Seongcheol Cho, Sungchan Kang, Tae Ho Yoon, Yoon Kim

**Affiliations:** 1 Department of Health Policy and Management, Seoul National University College of Medicine, 103 Daehak-ro, Jongno-gu, Seoul, 03080 Republic of Korea; 2 Big Data Steering Department, National Health Insurance Service, Wonju, Republic of Korea; 3 Department of Internal Medicine, Seoul Red Cross Hospital, Seoul, Republic of Korea; 4 Graduate School of Public Health, Seoul National University, Seoul, Republic of Korea; 5 Department of Preventive & Occupational Medicine, School of Medicine, Pusan National University, Pusan, Republic of Korea; 6 Institute of Health Policy and Management, Medical Research Center, Seoul National University, Seoul, Republic of Korea

**Keywords:** Geographic variation, Revascularization, Coronary artery bypass graft, Percutaneous coronary intervention, Beds

## Abstract

**Background:**

Korea has seen a rapid increase in the use of percutaneous coronary intervention (PCI) with the ratio of PCI to coronary artery bypass graft (CABG) the highest in the world. This study was performed to examine the factors associated with the rates of CABG and PCI.

**Methods:**

The data were acquired from the National Health Insurance database in Korea in 2013. We calculated the age-sex standardized rates of CABG and PCI. We examined the factors associated with the CABG and PCI rates by performing a regression analysis.

**Results:**

The rate of CABG showed a negative association with the deprivation index score, and other factors, such as the number of providers or hospital beds, did not show any significant association with the CABG rate. The rate of PCI had a strong negative association with the number of cardiothoracic surgeons and a strong positive association with the number of hospital beds.

**Conclusions:**

The positive association between the PCI rate and the number of hospital beds suggests that the use of PCI may be driven by the supply of beds, and the inverse association between the PCI rate and the number of cardiothoracic surgeons indicates the overuse of PCI due to lack of the providers of CABG. Policy measures should be taken to optimize the use of revascularization procedures, the choice of which should primarily be based on the patient’s need.

## Background

The choice of coronary artery bypass graft (CABG) and percutaneous coronary intervention (PCI) for the treatment of coronary artery disease has long been a debatable issue. The indications for PCI continued to expand with its technical advancement. This advancement of PCI, combined with its merit in terms of lower invasiveness and cost, resulted in the rapid augmentation of the volume of PCI and relative shrinkage of CABG [1]. The rise in the volume of PCI is a widespread phenomenon across the world. The two to eight folds ratio of rates of PCI to CABG has been documented worldwide [1–5]. However, the growth in the PCI use raises concerns for unnecessary or inappropriate use of PCI, which is highly probable in view of the large geographic variation in the rate of PCI and the PCI to CABG ratio [5, 6].

The use of CABG and PCI requires consideration of various elements such as the medical technology, costs, invasiveness, and patients’ preferences [7]. However, the choice should be made on the basis of what makes it optimal both in terms of patients’ health and health care costs. If the choice between the two procedures is determined by causes which are external to the patients’ health and are opposed to health care costs, it may not be in the best interest of the patients.

Korea has shown a prominent growth in the PCI rates and the ratio of the PCI to CABG rates. Between 2006 and 2016, the number of PCI increased by 66% while that of CABG decreased by 8% in Korea [8], and the ratio of PCI rates to CABG rates exceeded 20 in 2017 [9]. Even allowing for the recent dominance of PCI over CABG around the globe, the ratio of PCI over CABG rates in Korea is aberrantly high. The rapid growth in the use of PCI and high ratio of PCI over CABG rates are suggestive of the inappropriate utilization of PCI which was possibly affected by factors irrelevant to patients’ needs. Investigating the factors affecting the rates of CABG and PCI would elucidate the influence of factors such as the volume of resources and services. This study was performed to examine the factors associated with the rates of CABG and PCI. By using geographic variation study, we investigated the factors affecting the rates of the two procedures in terms of the service volume and provider density as well as the socio-economic circumstances of the regions.

## Methods

### Data

The cases of CABG and PCI were acquired from the National Health Insurance (NHI) database for the 2013 period. The NHI database contains health insurance claims data of the entire Korean population and provides claims data linked to information about patients’ demographics and health care facilities [10].

### Patient and public involvement

Patients and the public were not involved in the design or analysis of this study.

### Geographic variation in the rates of CABG and PCI

The utilization rate was defined as the number of procedures per 100,000 persons aged 20 and over. The rate was calculated based on the patients’ place of residence and age- and sex-standardized to the Korean resident population of 2013. We calculated the ratio of the 90th percentile to the 10th percentile (P90/P10), the coefficient of variation (CV), and the systematic component of variation (SCV). The SCV is a measure that estimates the variation between regions by removing the random part of variation due to within-region variation [10–12].

### Factors for geographic variation

We performed a linear regression with the rates of CABG and PCI as dependent variables. Utilization rates of CABG and PCI were calculated for 251 districts in Korea. The district is a widely used administrative unit for assessing the health care use by the population in Korea. It has an average population size of around 200,000, with large variation in its size, and has been considered a basic small-sized administrative unit for generating regional statistics and establishing policies [10]. However, the district may not necessarily correspond to the coverage area of all health care services, especially those requiring larger coverage areas. Although a smaller unit of analysis enables finer assessment of the areal characteristic in terms of demographic characteristics and statistical accuracy, a larger areal unit might be adequate to reflect the coverage area of the procedure. As CABG and PCI are the services mainly covered in tertiary level care, the characteristics concerning the use of those services need to be measured in a broader areal unit. Therefore, we divided the independent variables into two areal levels: district and hospital service areas (HSA). The HSA is a combined areal unit constructed on the basis of hospital utilization patterns of the residents. Three criteria were used to generate the hospital service area: 1. Minimum score of localization index - the proportion of health care use by the residents in an area [13]: 40%, 2. Minimum population size: 150,000, 3. Maximum transportation time within a region by car: 60 min. We used the HSA organized using data for the acute hospitalizations in Korea which occurred during 2011 and 2015 [14].

A total of four variables generated at the district level were used. First, regarding socio-economic status of an area, we used the deprivation index. The deprivation index is a composite indicator for socio-economic deprivation of a region, and a higher score of it represents a higher degree of deprivation. The deprivation index in this study was generated on the basis of a total of 9 items: the proportion of single-person households, the proportion of female headed households, the proportion of households without car ownership, the proportion of households not living in an apartment, the proportion of households living below the minimum housing standard, the proportion of the population aged 35–64 with no high school diploma, the proportion of households heads aged 15–64 engaged in manual labor, the proportion of the population who were divorced, separated, or bereaved among those aged 15 or over, and the proportion of the population aged 65 or over [15–18]. The score of each item was standardized by a Z-score, and its weighted values were summated as a deprivation index. The entire distribution of the deprivation index scores which we used is described elsewhere [19]. Second, we used the log of population size of an areal unit. Third, the number of primary care physicians per 100,000 was used. The primary care physicians were defined as physicians in the clinics where the proportion of the visits with 52 simple and minor disease groups, suggested by the Korean government to foster primary care [20], was over the average (38.3%) of total clinics [21]. Lastly, the utilization rate of the counterpart service (CABG for PCI, and vice versa) was included.

For HSA level variables, we used four variables: First, to reflect the influence of the absolute volume of the procedure performed in an area, we used the number of procedures of each dependent variable (CABG or PCI). Second, the number of cardiothoracic surgeons in general hospitals (including tertiary hospitals) per 100,000 was used. For an accurate assessment of the impact of the supply of cardiothoracic surgeons on the rates of CABG and PCI, we included only those specializing in cardiovascular surgery. Third, we included the number of cardiologists in general hospitals (including tertiary hospitals) per 100,000. As the number of cardiothoracic surgeons and cardiologists by regions could be acquired only from the websites of about 330 hospitals, we used the number as of 2019. Finally, to assess the impact of the hospital bed supply independently of supply of cardiothoracic surgeons and internists, we used the number of beds in large sized hospitals with more than 300 beds per 1000 persons. The data were provided by the Health Care Resources & Service Information Report [22, 23]. SAS, version 9.3 (SAS Institute, Inc., Cary, NC, USA) and SPSS 23 (IBM Corporation, Armonk, NY, USA) were used for the analysis.

## Results

**Fig. 1:**
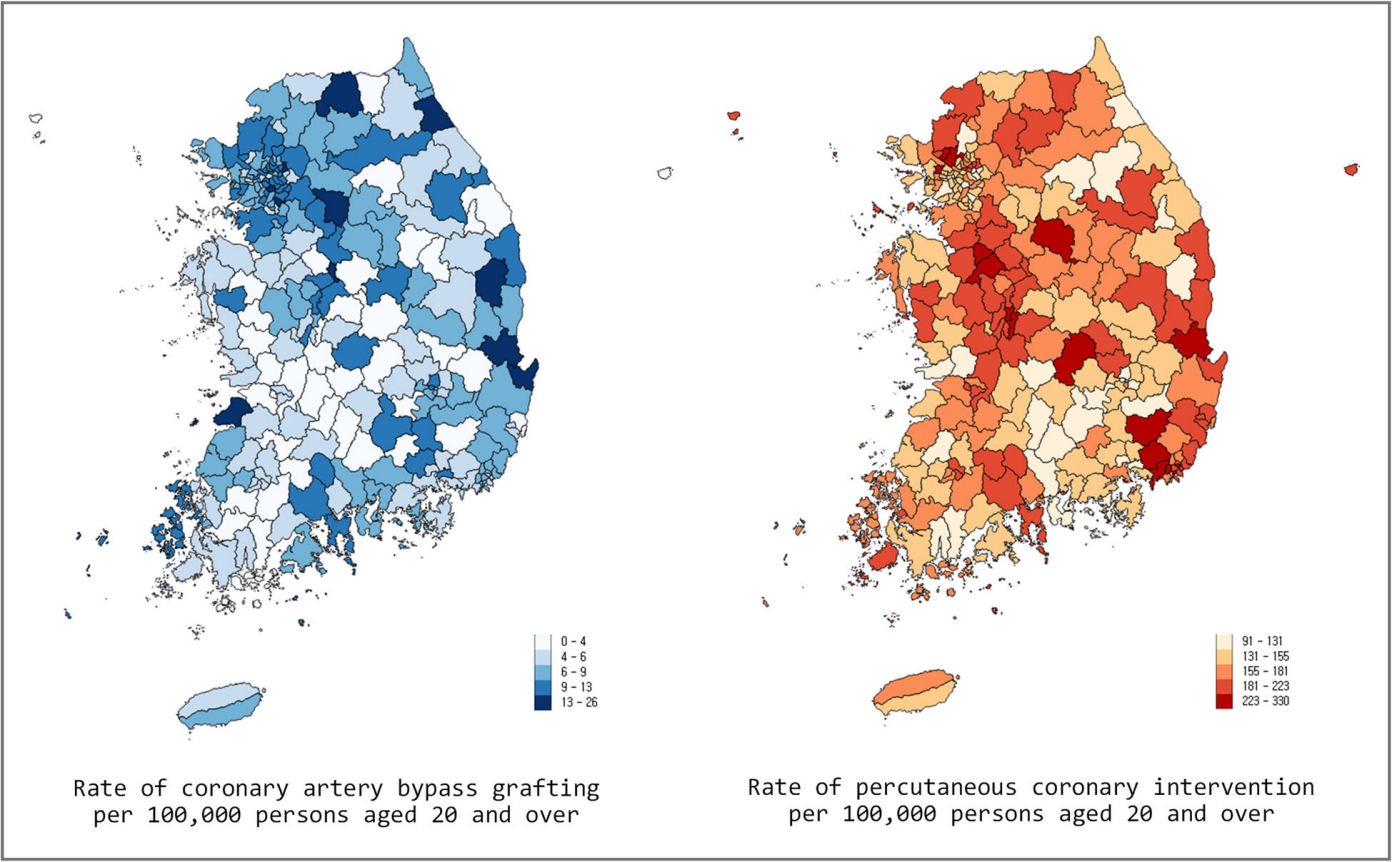
Map of the rates of coronary artery bypass grafting and percutaneous coronary intervention per 100,000 persons aged 20 and over

There were a total of 3086 CABGs and 68,452 PCIs among persons aged 20 and over in Korea in 2013 (Additional file 1 Table S1). The national rates of CABG and PCI were 7.7 and 170.3 per 100,000 persons aged 20 and over, respectively. Figure 1 presents a map of the rates of CABG and PCI at the district level. The CABG rate ranged from 0.0 to 30.7, and the PCI rate from 135.3 to 680.3. The variation statistics of CABG and PCI rates are presented in Table 1.

**Table 1:** Variation statistics of the rates of coronary artery bypass grafting and percutaneous coronary intervention

	CABG rate	PCI rate
National average	7.7	170.3
Standard deviation	3.3	36.5
Maximum	26.6	330.3
Minimum	0.0	91.3
P90/P10	3.2	1.7
CV	0.5	0.2
SCV (*100)	3.9	4.4

CABG and PCI rates are per 100,000 persons aged 20 and over*,* CABG *C*oronary artery bypass grafting*,* PCI *P*ercutaneous coronary intervention*,* CV *C*oefficient of variation*,* SCV *S*ystematic component of variation*,* P90/P10, ratio of the 90th to the 10th percentile of the distribution of rates. The rates were calculated for 100,000 persons aged 20 and over.

The results of the regression analyses showed a different pattern between the rates of CABG and PCI (Table 2). In the case of the CABG, only the deprivation index showed a statistically significant negative correlation with its rate. That is, as the degree of deprivation of a region increased, the CABG rate decreased. A one unit increase in the deprivation index score was associated with a 0.3 unit decrease in the CABG rate. Considering the distribution of the deprivation index scores (Additional file 1: Table S2), the shift in the deprivation index score from the lowest (least deprived) to the mean indicates a 3.2 unit decrease in the CABG rate, which is about 40% of the national average rate. Other factors, such as the supply of cardiothoracic surgeons, cardiologists, and hospital beds, and service volume did not show a significant association with the CABG rate. Regarding the PCI rate, the number of cardiothoracic surgeons showed a strong inverse association with the PCI rate, and the number of beds in general hospitals had a positive association. A one unit increase in the number of cardiothoracic surgeon was associated with a 65.5 unit decrease in the PCI rate. This indicates that the increase in the number of cardiothoracic surgeons from the lowest to the mean (Additional file 1: Table S2) is related to a 39.3 unit decrease in the PCI rate, 23% of the national average rate. A one unit increase in the number of beds in general hospitals was associated with a 59.4 unit increase in the PCI rate. This indicates that the increase in hospital beds from the lowest to the mean can be associated with a 101.0 unit increase in the PCI rates, about 60% of the national average rate. The number of cardiologists and the service volume had no significant association with the PCI rate.

**Table 2:** Regression analysis of CABG and PCI rates

Variables	CABG rate			PCI rate		
	Coefficient	95% CI		Coefficient	95% CI	
		Lower bound	Upper bound		Lower bound	Upper bound
Baseline (intercept)	9.095	.434	17.756	170.672	−4.021	345.365
District-level						
Deprivation index	−.272**	−.457	−.087	3.077	−.403	6.556
Log (population size)	−.378	−2.085	1.329	−5.943	−37.995	26.109
No. of primary care physicians per 100,000	.007	−.025	.039	.118	−.244	.480
PCI rate (CABG rate^1^)	.002	−.010	.013	−1.478	−4.462	1.506
Hospital service area-level						
No. of CABG (PCI^2^)	.001	.000	.002	−.002	−.004	.000
No. of cardiothoracic surgeons in general hospitals per 100,000	−.354	−2.054	1.346	−65.534**	− 106.809	− 24.260
No. of cardiologists in general hospitals per 100,000	.141	−.836	1.117	2.644	−18.122	23.411
No. of beds in general hospitals per 1000	−.386	−1.090	.317	59.406***	37.910	80.902
R^2^	.115			.402		

**p < 0.01, ***p < 0.001, CABG and PCI rates are per 100,000 persons aged 20 and over. 1. For the regression analysis with the dependent variable of PCI rate, CABG rate was used as an independent variables. 2. For the regression analysis with the dependent variable of PCI rate, the number of PCI in the hospital service areas was used

## Discussion

This study investigated geographic variation in rates of CABG and PCI in Korea and its factors using the 2013 National Health Insurance Database in Korea. The overall rates of CABG and PCI were 7.7 and 170.3 per 100,000 persons aged 20 and over, respectively. While only the deprivation index score showed a negative association with the CABG rates, the number of cardiothoracic surgeons and that of the beds in general hospitals had a negative and positive association with the PCI rates respectively.

The national rates of CABG and PCI in Korea are at a lower level compared with other countries [1–3, 5, 6, 24]. This is attributable to the relatively lower prevalence of ischemic heart disease in Korea [25]. However, compared with the PCI rate, the rate of CABG in Korea is at the markedly lowest level. As a result, the PCI to CABG ratio in Korea is at the highest level at more than 20 folds. Allowing for the global tendency of increase in the PCI to CABG ratio, which has reached even to eight in some countries [2, 26], the predominance of PCI over CABG in Korea is unusual. Our further analysis provides some explanation for this phenomenon.

The only significant factor for the CABG rate in this study was deprivation index score, which showed that the CABG rate was lower in more deprived areas. Considering the higher prevalence of ischemic heart disease in more deprived areas and higher risk of heart disease among the disadvantaged population in Korea as well as in other countries [5, 27, 28], the negative relationship between the CABG rate and the degree of deprivation seems contradictory. However, the negative association between revascularization rate and income of a region, even after adjusting for the burden of ischemic heart disease, has been reported [29]. Meanwhile, the deprivation index was not a significant factor for the PCI rate. This suggests that the use of CABG, which is more expensive than PCI, is not likely to be determined by the need of the population but rather by the ability to pay. The fact that the other supply-related variables showed no significant association with the CABG rate, as well as the negative association between the CABG rate and regional deprivation, supports the strong influence of the ability to pay on CABG use. In Korea, the out-of-pocket (OOP) expense of CABG is around $7000, which is more than 6 times that of PCI. Therefore, the high expense of the CABG could have been a cost barrier for persons in less affluent conditions. Considering that a considerable number of CABG’s are based on the referral, it is also likely that persons in disadvantaged conditions are not recommended for CABG as a treatment option at the beginning.

The factors, which showed statistically significant associations with the PCI rates, were the number of cardiothoracic surgeons and number of beds in general hospitals. The negative association between the number of cardiothoracic surgeons and PCI rates indicates that the supply of the providers of its counterpart procedure can have a decreasing effect on the PCI use. The higher PCI rates in regions with less cardiothoracic surgeons suggests two possibilities: the overuse of PCI in regions with less curbs from cardiothoracic surgeons or the inevitable use of PCI in regions with less cardiothoracic surgeons. The line of indications between CABG and PCI has become blurry, and cardiothoracic surgeons and cardiologists were driven to compete for the same patients [30, 31]. What matters is what determines the choice. If the decision is determined by the availability of relevant providers rather than patients’ needs, that might not necessarily be consistent with the patients’ best interests.

The positive association between the number of beds and the PCI rates substantiates the concern that the use of PCI may be induced by the supply factor. While earlier studies had found no association between the density of cardiologists and revascularization rates [29, 32], the influence of the density of hospital beds remained hardly investigated. Our result that only the number of beds, not that of cardiologists, showed a positive relationship with the PCI rates suggests that the hospital volume rather than an individual cardiologist is a strong factor for increasing the number of procedures. Higher availability of hospital beds, linked with the shortened waiting time [33], can increase or at least precipitate the use of PCI, and this tendency can be enhanced with the economic incentives. A finding from a prior study that the recommendation of the physician performing the diagnostic catheterization and the treating hospital are strong predictors of the mode of revascularization [34] indicates that the decision for the use of PCI involves considerable discretion. Elective PCI can be especially sensitive to hospital bed supply and economic incentives as it has more room for those two factors to intervene. According to a recent study in the US, half of elective PCI procedures were deemed uncertain or inappropriate while only 1% of the acute PCI procedures were [35]. Considering the proportion of elective PCI in Korea is 66%, which is twice that of the US [36], the inappropriate use of PCI is likely to be more prevalent, and the “beds to be filled” can be a main driver that facilitates elective PCI, a considerable portion of which are likely to be inappropriate.

There are a number of points to be addressed concerning the interpretation of the study results. First, as this is a cross sectional study, the relationship among variables cannot be considered a causal one, which should be further established by temporal analysis. However, the association revealed in this study would contribute toward unveiling the influence of supply factors for the PCI rates as well as CABG rates. Second, despite the inordinately high ratio of PCI to CABG in Korea, the absolute rate of PCI in Korea is still at a lower bracket. Therefore, to examine how much the PCI use in Korea is deviated from the appropriate amount, the prevalence and disease burden of cardiovascular disease should be taken into account. Third, as the unit of analysis in this study was a region, the individual factors which could affect the use of CABG and PCI could not be taken into account. However, though specified health factors were not accounted for, the age- and sex- adjusted utilization rates may serve as reliable indicators with due consideration of the patient factors which make the regional health care use differ.

## Conclusion

The PCI to CABG ratio in Korea is over 20, which is more than twice the highest ratio ever documented in the world. The high ratio of PCI to CABG in Korea needs to be addressed due to the concern of over-utilization of PCI as well as under-utilization of CABG. Our study results that the PCI rate is positively associated with the number of hospital beds suggest that the use of PCI may be driven by the supply of beds, and the inverse association between the PCI rate and the number of cardiothoracic surgeons indicates the overuse of PCI due to lack of providers of CABG. In contrast to the PCI rate which showed significant relationships with supplier factors, the CABG rate showed a positive association with the degree of regional deprivation. These results, considering the adjustment of the relevant supplier factors, imply the possibility that CABG might be under-utilized in the regions with lower socioeconomic conditions. Policy measures should be taken to optimize the use of revascularization procedures, the choice of which should primarily be based on the patient’s needs.

## Supplementary information

**Additional file 1: Table S1.** Absolute frequency of coronary artery bypass graft and percutaneous coronary intervention according to diagnostic groups. **Table S2.** Characteristics of the independent variables.

## Data Availability

The data are available from the authors.

## Abbreviations

CABG: Coronary artery bypass graft
CV: Coefficient of variation
HSA: Hospital service areas
NHI: National Health Insurance
P90/P10: 90th percentile to the 10th percentile of the distribution of cesarean section rates
PCI: Percutaneous coronary intervention
SCV: Systematic component of variation
