# Supplementary material for: Factors associated with the rates of coronary artery bypass graft and percutaneous coronary intervention
Source: BMC Cardiovasc Disord. 2019 Nov 29;19:275. doi: 10.1186/s12872-019-1264-3 (PMC6884838; doi:10.1186/s12872-019-1264-3)
Supplement: Supplementary file 1 — Additional file 1: Table S1. Absolute frequency of coronary artery bypass graft and percutaneous coronary intervention according to diagnostic groups. Table S2. Characteristics of the independent variables. [file 12872_2019_1264_MOESM1_ESM.docx]

Additional Table 1. Absolute frequency of coronary artery bypass graft and percutaneous coronary intervention according to diagnostic groups

| Age group | CABG | | PCI | | Population | |
| --- | --- | --- | --- | --- | --- | --- |
|  | Male | Female | Male | Female | Male | Female |
| 0-14 | 3 | 0 | 2 | 0 | 3,843,935 | 3,580,855 |
| 15-19 | 5 | 1 | 0 | 0 | 1,798,839 | 1,616,959 |
| 20-24 | 7 | 2 | 6 | 1 | 1,799,344 | 1,600,533 |
| 25-29 | 9 | 2 | 45 | 0 | 1,647,654 | 1,532,894 |
| 30-34 | 18 | 7 | 210 | 8 | 2,082,412 | 1,998,195 |
| 35-39 | 27 | 10 | 627 | 41 | 2,002,764 | 1,922,432 |
| 40-44 | 56 | 11 | 1,682 | 103 | 2,340,277 | 2,252,723 |
| 45-49 | 125 | 27 | 3,152 | 343 | 2,191,870 | 2,119,961 |
| 50-54 | 211 | 30 | 5,603 | 844 | 2,223,811 | 2,170,646 |
| 55-59 | 304 | 55 | 7,138 | 1,433 | 1,806,480 | 1,803,349 |
| 60-64 | 353 | 99 | 6,943 | 2,127 | 1,213,068 | 1,265,966 |
| 65-69 | 412 | 164 | 7,113 | 3,121 | 939,632 | 1,038,946 |
| 70-74 | 399 | 197 | 6,737 | 4,738 | 792,819 | 1,002,819 |
| 75+ | 323 | 238 | 7,699 | 8,738 | 853,382 | 1,596,007 |
| Total | 2,252 | 843 | 46,957 | 21,497 | 25,536,287 | 25,502,285 |
| National rate(>20) | 7.7 | | 170.3 | |  |  |
| National rate(>40) | 11.7 | | 263.0 | |  |  |
| National rate(>65) | 27.8 | | 610.5 | |  |  |
| National rate(all ages) | 6.1 | | 134.1 | |  |  |

Additional Table 2. Characteristics of the independent variables

| Variables | Mean | Minimum | Maximum | Standard deviation |
| --- | --- | --- | --- | --- |
| District-level |  |  |  |  |
| Deprivation index | .0 | -10.5 | 7.2 | 73.6 |
| Log (population size) | 5.1 | 4.0 | 5.8 | .4 |
| No. of primary care physicians per 100,000 | 29.8 | 5.0 | 140.0 | 14.9 |
| PCI rate (CABG rate) | 167.3 (7.1) | 91.3 (.0) | 330.3 (25.6) | 36.4 (3.3) |
| Hospital service area-level |  |  |  |  |
| No. of CABG (PCI) | 218.3 (7067.7) | .0 (166.0) | 1676.0 (16430.0) | 499.7 (6041.5) |
| No. of cardiothoracic surgeons in general hospitals per 100,000 | 0.6 | .0 | 2.0 | 0.5 |
| No. of cardiologists in general hospitals per 100,000 | 1.4 | .0 | 4.3 | 1.0 |
| No. of beds in large sized hospitals per 1,000 | 1.7 | .0 | 4.0 | 0.9 |

CABG and PCI rates are per 100,000 persons aged 20 and over
